# Histo-pathological patterns in hysterectomy specimens at a tertiary care centre in India

**DOI:** 10.6026/97320630019460

**Published:** 2023-04-30

**Authors:** Anjali Pandey, Meena Anil, Smriti Pandey

**Affiliations:** 1 Department of Pathology, Government Medical College, Ratlam, Madhya Pradesh, India

**Keywords:** Histo-pathological patterns, hysterectomy specimens, tertiary care centre

## Abstract

It is of interest to document histo-pathological patterns in hysterectomy specimens at tertiary care centre in India. This study included 442 cases. In this study, leiomyoma (9.17 %) was the most common preoperatively clinical diagnosis made in hysterectomy specimen. In this study, uterine fibroid showed a 90.47% correlation between pre-operative and histological findings. There was a 50 % correlation noted between adenomyosis and endocervical polyp.

## Background:

Hysterectomy is one of the most common and widely acceptable gynaecological surgical procedures performed worldwide. Histological examination is inevitable of the uterine specimen after hysterectomy [1]. Charles Clay performed the first sub-total hysterectomy in 1843 and the first total hysterectomy in 1929 in England [2]. Indications of gynaecological hysterectomy include uterine fibroid, adenomyosis, uterine prolapsed, abnormal uterine bleeding, prophylaxis against uterine cancer, malignancy of female reproductive organs, etc. [3]. The endometrium and myometrium of the uterus are influenced by different types of hormones periodically. The uterus, cervix, fallopian tube, and ovary are prone to crop up various non-neoplastic and neoplastic diseases. All these diseases are noticed across all age groups and contribute significantly to increased morbidity and mortality among women [4]. Dysfunctional uterine bleeding (DUB) or abnormal uterine bleeding (AUB), uterine prolapsed, uterine fibroid, adenomyosis, and endometriosis are common reasons for hysterectomy and practiced treatment of choice when other options are not available or have failed, or the patients had completed her family [5]. The incidence of a gynaecological hysterectomy varies from country to country, and it is an important issue in debates on medical ethics and health care across India [6]. In India, young women with low or no education who are undergoing a hysterectomy may have severe ill-health effects on their physical, reproductive, and socio-psycho health. Under the age of 40 years, little more than 1/3rd of women had a hysterectomy [7]. The purpose of the present study was to determine the histological pattern of hysterectomy specimens, associated clinical findings, and the correlation between pre-operative clinical and histological diagnosis at Government Medical College (GMC) Ratlam, a tertiary referral centre in Madhya Pradesh India. We discussed lesions, including non-neoplastic lesions, premalignant lesions, and malignant lesions in hysterectomy specimens received in the histopathology laboratory of our institute. Therefore, it is of interest to account for the pattern of clinical and histological lesions of hysterectomy among women in Ratlam domain Madhya Pradesh India.

##  Material and Methods:

The present study was carried out in the histology section of the department of pathology, Government Medical College (GMC) Ratlam. Ethical approval for our research (Ethical Committee No.-GMC/Ratlam/2020/IEC/003/15/06/2020) was provided by the Ethical Committee of Government Medical College Ratlam Madhya Pradesh (M.P.), on the date 15 June 2020. A one-year retrospective study of hysterectomy specimens from February 2019 to January 2020 was carried out. During this one year, 439 uterine samples were obtained from the Obstetrics and Gynaecology department of GMC Ratlam. Inclusion criteria were that all hysterectomy specimens receive with complete histo-pathological requisition form of patients. Hysterectomy specimens with incomplete histopathological requisition forms and obstetrical hysterectomies were excluded from the study. A record of patients was retrieved; age, presenting symptoms, clinical details, parity, sonographic findings, and indications of hysterectomy were recorded retrospectively. All the specimens were fixed in 10% formalin and tissue sections were taken from a representative area for processing. Subsequently, the tissues were dehydrated with ascending grades of alcohol, clear in xylene, and embedded in paraffin. Thereafter, 3-5 microns thick paraffin sections were cut on a rotary microtome, dewaxed, and stained with hematoxylin and eosin (H and E) stain. Histological diagnoses were recorded and Data was analyzed percentage-wise.

## Results:

This study included 442 cases. The age ranges of those who had hysterectomies for various reasons were from 18- 80 years (Table 1 ].

In this study, leiomyoma (9.17 %) was the most common preoperatively clinical diagnosis; whereas, Dysfunctional uterine bleeding/ Abnormal uterine bleeding (DUB/AUB) (8.21%) was the second most common diagnosis and least common lesions 0.48% diagnosed as pyometra and cervical polyp as shown in the Figure below.

Various types of hysterectomy specimen included in the study, which was shown in the Table 2.

The most frequent lesions seen in the cervix were 425 (96.1 %), the next common being endometrium 415 cases (93.8%), myometrium 407 (92.08%) cases, ovary 171 (38.6 %) and fallopian tube 103 (23.3 %). A total of 10 cases of Squamous cell carcinoma were noted, of which 3 were well differentiated, 4 were moderately differentiated, 2 cases showed poorly differentiated, and 1 with basaloid differentiation. Detailed histological lesions reported in the specimens given in the table below (Table 3).

**Figure 2:**
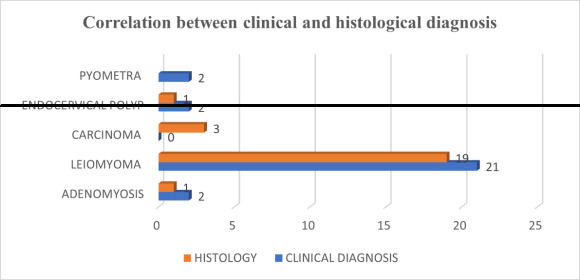
Showing correlation between clinical and histological diagnosis

In this study, uterine fibroid showed a 90.47% correlation between pre-operative and histological findings. There was a 50 % correlation noted between adenomyosis and endocervical polyp. Whereas, 2 cases clinically diagnosed as pyometra reported histologically papillary endocervicitis and endocervical polyp respectively. (Figure 2)

## Discussion:

In India, hysterectomy contributes to 6% of all surgical procedures done. [8] The current study was a one-year retrospective study and represents various lesions seen histologically in hysterectomy specimens and their clinical correlation. In this study, 442 cases were included with age ranges from 18-80 years with the commonest age group between 31 - 40 years (203 cases, 45.9%), followed by 41 - 50 years (144 cases, 32.57 %). Various studies also reported the commonest age group between 41 - 50 years, such as Yadav *et al* [9] (52.38%), Dhuliya *et al* [10] (48%), and Chavhan *et al*. [11] (50 %). Whereas, Arunadevi *et al*. [12] showed the commonest age group between 40 - 49 years in 48.31 % and 22.5 % cases, respectively. The most common indication for hysterectomy in our study was leiomyoma (38 cases, 9.17 %), followed by DUB/AUB (34 cases, 8.21 %). This is similar to the study done by Yadav *et al*,[9] which also showed leiomyoma (48.57%) and DUB (25.71%) causes of hysterectomy. Regarding, the most common preoperative procedure performed in the present study was Total hysterectomy (TH) without salpingo-oophorectomy (SOP) (310 specimens, 70.1%), after that TH with unilateral SOP (58 specimens, 13.1%) and TH with bilateral SOP (46 specimens, 10.4%). A study was done by Mallapa *et al*.[13] on 238 cases, included Pan abdominal hysterectomy (71%) as the most common hysterectomy procedure, followed by Vaginal hysterectomy (16.8%) and TAH (10.92%). Our study reported the majority of lesions were in the cervix (425 cases,96.15%) then endometrium (415 cases,93.8%), and later myometrium (407 cases, 92.08%), fallopian tube (103 cases, 23.3%), ovary (171 cases, 38.6%).Whereas, the most common lesions reported by Sreedhar *et al*.[14] was in the endometrium (84 cases, 42%), myometrium (59 cases, 29.5%), consequently cervix (33 cases, 16.5%), and ovary (24 cases, 2%). Various commonest lesions were seen in the different studies shown in the table below. (Table 4)

The present study reported 10 (2.35%) cases of Squamous cell carcinoma in the cervix, 13 (0.72%) uterine malignancy, and 3 malignant cases in the ovary that included 0.58 % yolk sac tumor, and 1.06% of krukenberg tumor.Baral *et al*. [ 18] only reported ovarian malignancy in which 1.2% and 0.58% were serous cystadenocarcinomata and krukenberg tumors, respectively. Ahmed *et al*. [19] noticed significant carcinoma cases in hysterectomy specimens, i.e. 25% of cases were of endometrium carcinoma, in which 61% was adenocarcinoma and 39% had stromal sarcomas. This study also showed 1077 cervical lesions, in which 82% of cases had Squamous cell carcinoma, 9% had adenocarcinoma, and 4.5% each of adenosquamous and carcinosarcoma cases. In our study correlation between clinical and histological diagnosis for leiomyoma was 90.47%, followed by 50% for adenomyosis. This finding is correlating with the Gupta *et al*. [20] with 95.83% and 66.67 % correlation with leiomyoma and adenomyosis subsequently. In contrast, to these studies, Perveen *et al*. [21] reported a 90% and 65 % correlation between adenomyosis and leiomyoma. Sarwar *et al*. [22] also reported a 70 % clinical-histological correlation for malignancy and 47.1%, 28%, and 16.6% for leiomyoma, adenomyosis, and endometrial polyp respectively

## Conclusion:

Leiomyoma and AUB/DUB is the most common indication for hysterectomy. In our study, adenomyosis is the most common histological finding seen in the specimens. When a fibroid diagnosis was made clinically on a specimen, there was a strong association. The poor association is seen with malignant cases. Therefore, every hysterectomy specimen needs to go through an Histopathological examination to confirm the diagnosis and for delivering the best possible care for specific cancer.

## Figures and Tables

**Figure 1:**
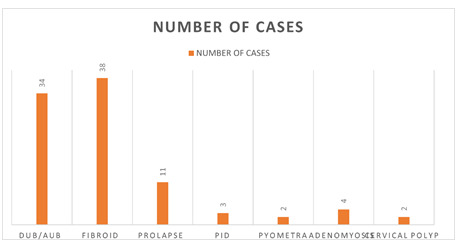
Chart showing various causes for hysterectomies

**Table 1:** Age group distribution among the cases

**Age range (years)**	**Number of cases **	**Percentage (%)**
<20	1	0.22
20 - 30	54	12.2
31 - 40	203	45.9
41 - 50	144	32.57
51 - 60	32	7.2
61 - 70	5	1.1
71 - 80	3	0.67
Total	442	100

**Table 2:** Types of Hysterectomy specimens

**Type of specimen**	**Number of specimen **	**Percentage (%)**
Total hysterectomy without SOP	310	70.1
Total hysterectomy with bilateral SOP	46	10.4
Total hysterectomy with unilateral SOP	58	13.1
Oophorectomy	18	4.07
Polypectomy	2	0.45
Endometrial biopsy	8	1.8
Total	442	100

**Table 3:** Histological lesions of Hysterectomy specimens

**Anatomical site **	**Type of lesions**		**No. of cases **	**Percentage (%)**
Cervix	Chronic cervicitis		164	38.5
	Papillary endocervicitis		73	17.1
(n= 425)	Endocervical polyp		15	3.5
	Squamous metaplasia		13	3.05
	LSIL		61	14.3
	HSIL		34	8
	SCC		10	2.35
Endometrium	Phase of endometrium			
	Proliferative		132	31.8
	Secretory		194	46.7
(n= 415)	Biphasic		2	0.48
	Endometrial polyp		28	6.74
	Endometritis		26	6.26
	Decidual reaction		11	2.65
	Senile changes		34	8.19
	Simple hyperplasia without atypia		1	0.24
	Simple hyperplasia without atypia		0	0
	Complex hyperplasia without atypia		3	0.7
	Complex hyperplasia with atypia		1	0.2
	Malignant tumors		2	0.48
	Inadequate for opinion		1	0.2
Myometrium	Fibroid			
	Intramural		53	13
(n= 407)	Submucosal		24	5.8
	Subserosal		9	2.21
	Secondary changes		10	2.45
	Adenomyosis		175	42.9
	Monckeberg sclerosis		2	0.49
	Malignancy		1	0.24
	Normal histology		229	56.2
Fallopian tube	Hydrosalpinx		7	6.79
	Hematosalpinx		2	1.94
(n= 103)	Paratubal cyst		2	1.94
	Ghost chorionic villi		1	0.97
	Normal histology		92	89.32
0vary	Foliicular cyst	Right	1	0.58
(n=171)		Left	0	0
	Corpus luteal cyst	Right	1	0.58
Right (n=120)		Left	3	1.75
Left (n=51)	Simple serous cyst	Right	16	9.35
		Left	5	2.92
	Serous cystadenoma	Right	20	11.6
		Left	7	4.09
	Mucinous cystadenoma	Right	1	0.58
		Left	1	0.58
	Hemorrhagic cyst	Right	2	1.16
		Left	0	0
	Tubercular	Right	2	1.16
		Left	0	0
	Chronic oophoritis	Right	1	0.58
		Left	0	0
	Mature cystic teratoma	Right	7	4.09
		Left	2	1.16
	Yolk sac tumor	Right	1	0.58
		Left	0	0
	Krukenberg tumor	Right	1	0.58
		Left	1	0.58
	Normal histology	Right	68	39.76
		Left	32	18.71

**Table 4:** Commonest lesions seen in different studies

**STUDY**	**NO. OF CASES **	**MOST COMMON LESIONS PRESENT**									
		CERVIX		ENDOMETRIUM		MYOMETRIUM		FALLOPIAN TUBE		OVARY	
Pandya et al. [15]	250	Chronic nonspecific cervicitis	50.40%	Atropic	6.80%	Leiomyoma	38.40%	-		Dermoid& simple serous cyst	17.10%
Titiloye et al. [16]	1086	Chronic nonspecific cervicitis	25%	Hyperplasia	67.70%	-	-	Salpingitis	3.83%	cyst	15.10%
Singh et al [17]	376	Chronic nonspecific cervicitis	81.95%	Polyp	4.50%	Leiomyoma	20.50%	Metastaisi/invasion	2%	Serous cystadenoma	5.40%
Present	442	Chronic nonspecific cervicitis	38.50%	Polyp	6.70%	Adenomyosis	42.90%	Hydrosalpinx	6.70%	Serous cystadenoma	15.60%
											
